# MKRMDA: multiple kernel learning-based Kronecker regularized least squares for MiRNA–disease association prediction

**DOI:** 10.1186/s12967-017-1340-3

**Published:** 2017-12-12

**Authors:** Xing Chen, Ya-Wei Niu, Guang-Hui Wang, Gui-Ying Yan

**Affiliations:** 1 School of Information and Control Engineering, China University of Mining and Technology, Xuzhou, 221116 China; 2 School of Mathematics, Shandong University, Jinan, 250100 China; 3 Academy of Mathematics and Systems Science, Chinese Academy of Sciences, Beijing, 100190 China

**Keywords:** miRNA, Disease, miRNA–disease association, Multiple kernel learning, Kronecker regularized least squares

## Abstract

**Background:**

Recently, as the research of microRNA (miRNA) continues, there are plenty of experimental evidences indicating that miRNA could be associated with various human complex diseases development and progression. Hence, it is necessary and urgent to pay more attentions to the relevant study of predicting diseases associated miRNAs, which may be helpful for effective prevention, diagnosis and treatment of human diseases. Especially, constructing computational methods to predict potential miRNA–disease associations is worthy of more studies because of the feasibility and effectivity.

**Methods:**

In this work, we developed a novel computational model of multiple kernels learning-based Kronecker regularized least squares for MiRNA–disease association prediction (MKRMDA), which could reveal potential miRNA–disease associations by automatically optimizing the combination of multiple kernels for disease and miRNA.

**Results:**

MKRMDA obtained AUCs of 0.9040 and 0.8446 in global and local leave-one-out cross validation, respectively. Meanwhile, MKRMDA achieved average AUCs of 0.8894 ± 0.0015 in fivefold cross validation. Furthermore, we conducted three different kinds of case studies on some important human cancers for further performance evaluation. In the case studies of colonic cancer, esophageal cancer and lymphoma based on known miRNA–disease associations in HMDDv2.0 database, 76, 94 and 88% of the corresponding top 50 predicted miRNAs were confirmed by experimental reports, respectively. In another two kinds of case studies for new diseases without any known associated miRNAs and diseases only with known associations in HMDDv1.0 database, the verified ratios of two different cancers were 88 and 94%, respectively.

**Conclusions:**

All the results mentioned above adequately showed the reliable prediction ability of MKRMDA. We anticipated that MKRMDA could serve to facilitate further developments in the field and the follow-up investigations by biomedical researchers.

**Electronic supplementary material:**

The online version of this article (10.1186/s12967-017-1340-3) contains supplementary material, which is available to authorized users.

## Background

MicroRNAs (miRNAs) are a class of endogenous and small noncoding RNAs, which function in RNA silencing and post-transcriptional regulation of gene expression via base-pairing with complementary sequences within mRNA molecules [1–6]. However, some researches have shown that in some cases miRNAs could also function as positive regulators [7, 8]. Since the first discovery of miRNAs (*C. elegans lin*-*4*) in the early 1990s, thousands of currently annotated miRNAs have been identified from a wide variety of species, ranging from nematodes to humans (for example, more than 1800 homo sapiens miRNAs according to miRBase21) [9–13]. In addition, plenty of evidences have shown that miRNAs play important roles in many fundamental and critical biological processes, such as cell growth, proliferation, differentiation, development, apoptosis, metabolism, aging, signal transduction, viral infection and so on [14–19]. Thus, it is not surprising that more and more miRNAs have been reported to be associated with various complex human diseases [20–22]. For example, compared with normal tissue controls as measured by microarray, miR-129, miR-142, and miR-25 were differentially expressed in every pediatric brain tumor type [23]. Furthermore, according to hepatitis C virus (HCV) case report, the miR-122 expression level could be down-regulated by HCV core protein in a time- and dose-dependent manner [24]. Moreover, compared with the healthy gingiva, in periodontitis cases, six miRNAs (let-7a, let-7c, miR-130a, miR-301a, miR-520d and miR-548a) were up-regulated more than eightfold [25]. Additionally, miR-372 and miR-373 were highly up-regulated in the cerebellar tumors compared with normal cerebellum or whole brain [26]. Therefore, identifying potential disease-related miRNAs could not only significantly contribute to comprehending the diseases mechanisms, but also be beneficial to the prognosis, diagnosis, treatment and prevention of human complex diseases [27–30]. However, as is known, traditional experimental methods are usually expensive and time-consuming. Fortunately, as the accumulated results of vast biology experiments, some reliable miRNA-related datasets have been constructed and updated. So it is necessary and viable to develop more efficient and feasible computational approaches to predict underlying diseases associated miRNAs based on available biological datasets. In addition, the promising predicted results obtained by computational methods could be used as guidance for further experimental validation [31, 32].

In fact, based on the hypothesis that functionally similar miRNAs are often associated with phenotypically similar diseases and vice versa [12, 33–37], many computational models have been proposed for predicting disease-associated miRNAs during the last years. For example, Jiang et al. [27] presented a network-based approach, which scored each miRNA in the miRNA network through the cumulative hypergeometric distribution to predict potential miRNA–disease associations. Considering the functional connections between miRNA targets and disease genes in protein–protein interaction (PPI) networks, Shi et al. [38] developed a computational method to identify miRNA–disease associations by performing random walk. Their model took advantage of human PPIs, the miRNA–target interactions and disease–gene associations to predict potential associations between the miRNAs and diseases based on the assumption that miRNAs could tend to be associated with diseases which have more correlated associations with the miRNA targets. By integrating protein–disease associations and miRNA–protein interactions, Mork et al. [39] presented a miRPD (miRNA–protein–disease) approach to predict novel miRNA–disease associations. In their model, they inferred disease–miRNA associations and ranked them according to a scoring scheme that combined the miRNA–protein association scores and protein–disease association scores. However, all the above three models strongly depended on the miRNA–target interactions with high rate of false-positive and high false-negative results. Chen et al. [40] presented the Random Walk with Restart for MiRNA–disease association (RWRMDA) model. In their method, they mapped all the miRNAs (containing seed miRNAs and candidate miRNAs) to miRNA functional similarity network. Then, they implemented random walk with restart until they got stable probability. Finally, they ranked all the candidate miRNAs based on the stable probability to select potential disease-related miRNAs for experimental validation. Meanwhile, that approach was the first global network-based method and it did not rely on predicted miRNA–target interactions. Xuan et al. [41] developed a HDMP method based on weighted *k* nearest neighbors. They calculated the miRNAs functional similarity matrix by incorporating the semantic similarity and the phenotype similarity between diseases. Then they adopted a unique weight assignment of miRNAs based on miRNA family or cluster. Finally, the relevance score of unlabeled miRNA with investigated disease was calculated by considering the functional similarities of its weighted k most similar neighbors and the distribution information of the labeled miRNAs in these neighbors. Considering that the simple similarity-based ranking of k-nearest-neighbors was not reliable for further prediction, Chen et al. [42] proposed a computational method of ranking-based KNN for miRNA–disease association prediction (RKNNMDA) to predict potential related miRNAs by reranking these previously similarity-based sorted neighbors for better prediction results. Li et al. [43] developed a matrix completion for MiRNA–disease association prediction (MCMDA) using matrix completion algorithm based on the known miRNA–disease associations to predict the potential miRNA–disease associations. Although the prediction performances of these mentioned approaches were pretty good, they could not be implemented for the diseases without known related miRNAs. Furthermore, HDMP strongly relied on the selection of the number of nearest neighbors considered in the model and it failed to set different values of this parameter when different diseases were investigated. Recently, Chen et al. [44] proposed the model of within and between score for MiRNA–disease association prediction (WBSMDA). WBSMDA integrated miRNA functional similarity, disease semantic similarity, known miRNA–disease associations, and Gaussian interaction profile kernel similarity for diseases and miRNAs into an integrated similarity for diseases and miRNAs respectively, then the model combined within-score and between-score from the view of miRNAs and diseases to calculate the association probability for miRNA–disease pairs. WBSMDA could be implemented for the diseases without known associated miRNAs. Then, Chen et al. [45] developed the computational model of Heterogeneous Graph Inference for MiRNA–disease association prediction (HGIMDA) by integrating known verified miRNA–disease associations, miRNA functional similarity, disease semantic similarity, Gaussian interaction profile kernel similarity into a heterogeneous graph. Then they could infer potential association between disease and miRNA by summarizing all paths with the length equal to three in the graph. Compared with previous computational models, HGIMDA model got a better prediction performance and could be effectively applied to new diseases and new miRNAs without any known associations, which overcame the important limitations of many previous computational models.

Additionally, some studies developed machine learning-based computational models to predict potential disease–miRNA associations. For example, according to the assumption that miRNAs associated with specific tumor phenotype would show aberrant regulation of their target genes, Xu et al. [5] proposed an approach based on the miRNA target-dysregulated network (MTDN) to prioritize potential diseases associated miRNAs. Based on the network topology information, some feature measures were extracted for miRNAs in MTDN. Then the authors used support vector machine (SVM) to construct classifier for distinguishing positive miRNA–disease associations from negative associations. Nowadays, by utilizing the network information flow model, Yu et al. [46] developed a combinatorial prioritization algorithm of maximizing network information flow (MaxFlow) to predict microRNA–disease associations based on the microRNAome-phenome network. To overcome the negative influence on model prediction performance that resulted from the selection bias of negative samples, Chen et al. [47] developed a computational model of regularized least squares for MiRNA–disease association (RLSMDA). RLSMDA model was implemented in the framework of a semi-supervised learning, which meant that it needed no negative samples. Recently, considering that no previous computational methods could predict the types of disease–miRNA associations, Chen et al. [48] developed the model of Restricted Boltzmann machine for multiple types of miRNA–disease association prediction (RBMMMDA). RBMMMDA model could obtain not only new miRNA–disease associations, but also the corresponding association types by employing Restricted Boltzmann machine (RBM). Predicting the different types of disease–miRNA associations could be beneficial for our understanding about the molecular basis of diseases in the level of miRNAs. RBMMMDA model is the first model that could infer association types of miRNA–disease pairs on a large scale.

Before presenting our model, we briefly introduced some information about kernel-based methods. Given a known disease–miRNA association network, kernel based methods could be implemented to predict unknown miRNA–disease interactions, where a kernel could be seen as a similarity matrix of miRNAs or diseases. Kernel based approaches used some base kernels, such as disease semantic similarity or miRNA functional similarity, to measure the similarity between diseases or miRNAs. Then, a pairwise kernel function, which measured the similarity between disease–miRNA pairs, could be calculated by combining a miRNA base kernel and a disease base kernel via kernel product. Multiple kernel learning (MKL) was a machine learning method focusing on the search for an optimal combination of base kernels [49]. However, since traditional MKL methods were based on SVM [49, 50], they were subject to memory limitations imposed by the pairwise kernel function and the difficulty of obtaining negative samples in supervised learning. Kronecker regularized least squares approach (KronRLS) [51] abandoned SVM and took advantage of the algebraic properties of Kronecker product to implement predictions without the explicit calculation of pairwise kernels function. However, KronRLS method could not be conducted to solve multiple kernels situations because it was initially developed to handle single kernel situation.

In this work, we proposed a computational approach named Multiple kernel learning-based Kronecker Regularized least squares for MiRNA–disease association prediction (MKRMDA). To this end, we extended the KronRLS method to a MKL scenario. Our method used L2 regularization to produce a finally optimized non-sparse combination of multiple base kernels, which was then used for the prediction process. Additionally, the proposed method could cope with large disease and miRNA association matrices. Furthermore, we implemented Leave-one-out cross validation (LOOCV) for MKRMDA. As a result, MKRMDA obtained a global AUC value of 0.9040 and a local AUC value of 0.8446, performing better than some previous models mentioned above, such as WBSMDA [44], HDMP [41], RLSMDA [47], HGIMDA [45], MCMDA [43], RKNNMDA [42] and MaxFlow [46]. Moreover, we carried out three different patterns of case studies in this work (more details in part 3.2). As mentioned in abstract, there were high ratios of the predicted miRNAs confirmed in all three ways of case studies by corresponding databases. Therefore, it showed the effectivity of MKRMDA in predicting potential miRNA–disease associations for various categories of diseases.

## Methods

### Human miRNA–disease associations

Human miRNA–disease associations dataset employed in this work were obtained from the HMDDv2.0 database [52], consisting of 5430 experimentally confirmed human miRNA–diseases associations about 495 miRNAs and 383 human diseases. We adopted the adjacency matrix *A* to clearly describe the known miRNAs–disease associations. Specifically, if miRNA *m*(*i*) was confirmed to be related to disease *d*(*j*), the entity *A*(*i,j*) was assigned 1, otherwise 0.

### MiRNA functional similarity

MiRNA functional similarity has been worked out previously by Wang et al. [35]. In this study, benefitting from their relevant researches, we downloaded the relevant miRNA functional similarity measures information from http://www.cuilab.cn/files/images/cuilab/misim.zip and constructed the corresponding miRNA functional similarity matrix *FS*, where *FS*(*i,j*) was denoted as the functional similarity score between miRNA *m*(*i*) and *m*(*j*). We got the known miRNA functional similarity about 271 miRNAs in this way. For the rest 224 miRNAs without known functional similarity, we calculated the Gaussian interaction profile kernel similarity, which would be introduced in part 2.5. By integrating the known 271 miRNA similarity entries and the 224 newly calculated Gaussian similarity entries, the miRNA similarity matrix had exact 495 entries for prediction work.

### Disease semantic similarity model 1

Based on the disease MeSH descriptor downloaded from the National Library of Medicine (http://www.nlm.nih.gov/), the relationship between different diseases could be represented by a structure of directed acyclic graph (DAG). For an arbitrary disease *D*, DAG(*D*) = (*D*, T(*D*), E(*D*)) can be defined to represent the disease *D*, where T(*D*) is a node set, consisting of *D* itself and all its ancestor nodes, E(*D*) is the corresponding edge set, consisting of the directed edges pointing from parent nodes to child nodes [35]. The semantic value of disease *D* could be defined as follows:

1 $${\text{DV}}1\left( D \right) = \mathop \sum \limits_{d \in T(D)} D1_{D} \left( d \right)$$

2 $$\left\{ \begin{aligned} D1_{D} \left( d \right) & = 1\quad \quad \quad \quad \quad \quad \quad \quad if\;d = D \\ D1_{D} \left( d \right) & = max\left\{ {\Delta_{*} D1_{D} \left( {d^{\prime}} \right)|d^{\prime}\; \in \;child\;of\;\left. d \right\}} \right. \\ if\,d & \ne D \\ \end{aligned} \right.$$

where $\Delta$ is the semantic contribution factor. It is obvious that for a given disease *D*, as the distance between *D* and another disease, *d*, increases, the contribution score of *d* for disease *D* decreases. In this method, diseases located in the same layer would contribute the same score to the semantic value of disease *D*. Finally, the semantic similarity between disease *d*(*i*) and *d*(*j*) can be calculated based on the observation that two diseases with larger common part of their DAGs will have larger similarity score:

3 $${\text{SS}}1\left( {d\left( i \right),d\left( j \right)} \right) = \frac{{\mathop \sum \nolimits_{t \in T(d(i))\mathop \cap \nolimits T(d(j))} (D1_{d(i)} \left( t \right) + D_{1d(j)} (t))}}{{DV1\left( {d\left( i \right)} \right) + DV1(d(j))}}$$

where SS1 represents the disease semantic similarity matrix in this model.

### Disease semantic similarity model 2

In this calculation method of disease semantic similarity, different from the above method, we assign different contribution value to the diseases in the same layer of DAG(*D*) out of the consideration that disease which appears in less DAGs contributes to the semantic similarity of disease *D* at a higher contribution level. So the contribution of disease *d* in DAG(*D*) to the semantic value of disease *D* is defined as follows when *nd* represent the number of all diseases and ${\text{DAG}}_{\text{t}}$ represents the number of DAGs including t:

4 $$D2_{D} \left( d \right) = - \;{ \log }\left( {\frac{{DAG_{t} }}{nd}} \right)$$

Then, the semantic similarity of disease *d*(*i*) and *d*(*j*) can be calculated as follows:

5 $${\text{SS}}2\left( {d(i),{\text{d}}({\text{j}})} \right) = \frac{{\mathop \sum \nolimits_{t \in T(d(i))\mathop \cap \nolimits T(d(j))} (D2_{d(i)} \left( t \right) + D2_{d(j)} \left( t \right))}}{{DV2\left( {d(i)} \right) + DV2(d(j))}}$$

where SS2 represents the disease semantic similarity matrix in this model.

### Gaussian interaction profile kernel similarity

Gaussian kernel function is a kind of widely used radial basis function (RBS), based on which the Gaussian interaction profile kernel similarity could be calculated by taking advantaging of the known miRNA–disease association information. Specifically, by observing whether a disease *d*(*i*) is associated with each miRNA or not, binary vector *IP*(*d*(*i*)), the *i*th column of the adjacency matrix A, could be obtained and denoted as the interaction profiles of disease *d*(*i*). Then, Gaussian kernel similarity between disease *d*(*i*) and *d*(*j*) can be calculated as follows:

6 $$GD\left( {d\left( i \right),d\left( j \right)} \right) = exp\left( { - \gamma_{d} \parallel {IP\left( {d\left( i \right)} \right) - IP(d\left( j \right))}\parallel^{2} } \right)$$

where $r_{d}$ is adopted to control the kernel bandwidth, *GD* represent Gaussian interaction profile kernel similarity of diseases. In addition, $r_{d}$ can be obtained by normalizing a new bandwidth parameter $r^{\prime}_{d}$ by the average number of known associations with miRNAs per disease as follows:

7 $$r_{d} = {{r^{\prime}_{d} } \mathord{\left/ {\vphantom {{r^{\prime}_{d} } {\left( {\frac{1}{nd}\mathop \sum \limits_{i = 1}^{nd}  IP\left( {d\left( i \right)} \right)^{2} } \right)}}} \right. \kern-0pt} {\left( {\frac{1}{nd}\mathop \sum \limits_{i = 1}^{nd} \parallel{IP\left( {d\left( i \right)} \right)\parallel }^{2} } \right)}}$$

where *nd* is denoted as the number of all the diseases investigated. In principle, the new bandwidth parameter $r^{\prime}_{d}$ could be set with cross-validation, but in this article, $r^{\prime}_{d}$ was set 1 based on previous studies [53, 54].

Additionally, the construction method of miRNA Gaussian interaction profile kernel similarity matrix, *GM*, is similar to the calculation of disease Gaussian interaction profile kernel similarity:

8 $$GM\left( {m\left( i \right),m\left( j \right)} \right) = exp \left( { - \gamma_{m}\parallel{ IP(m\left( i \right)) - IP(m(j))\parallel}^{2} } \right)$$

9 $$\upgamma_{\text{m}} = \frac{{{\gamma^{\prime}}_{m} }}{{\left( {\frac{1}{nm}\mathop \sum \nolimits_{{{i} = 1}}^{nm}\parallel{ {IP}\left( {m\left( {i} \right)} \right)}\parallel^{2} } \right)}}$$

where *nm* is denoted as the number of all the miRNAs investigated.

### MKRMDA

With the advance of sequencing technology and biology, more and more reliable biological data about disease and miRNA had been released, including various similarity information about disease and miRNA. If we could efficiently take advantage of the multi-source similarity data as more as possible, we could obtain more precise information about disease–miRNA associations. Hence, in this work, we proposed the MKRMDA to predict potential disease associated miRNAs in the situation where multiple kernels were involved, meaning that much more similarity information could be integrated. To this end, at first we briefly introduced the relevant classification algorithm, which could be used in single kernel problem. Given a set of diseases ${\text{D}} = \left\{ {d(1),d(2) \ldots ,d(nd)} \right\}$, a set of miRNAs ${\text{M}} = \left\{ {m(1),m(2) \ldots ,m(nm)} \right\}$, we could obtain a set of training samples $S = \left\{ {\left( {x_{1} ,y_{1} } \right),\left( {x_{2} ,y_{2} } \right) \ldots \left( {x_{n} ,y_{n} } \right)} \right\}$, $x_{i}$ represented a disease–miRNA pair, and $y_{i}$ represented the corresponding binary labels, where 1 stood for a known association and 0 otherwise with $1 < i \le n,n = nd \times nm$, which meant the number of all disease–miRNA pairs. In our model, if a miRNA–disease pair $x_{i}$ was a known miRNA–disease association recorded in HMDDv2.0 database, the corresponding $y_{i}$ was set 1, otherwise 0. Denoting the training set as S, our goal was to learn a function *f* that could generalize well on new samples, namely new disease–miRNA pairs. Then this problem could be solved based on the closely related (via Lagrange multipliers) Tikhonov minimization problem as follows [55]:

10 $$\mathop {\hbox{min} }\limits_{f \in H} \frac{1}{n}\mathop \sum \limits_{i = 1}^{n} V\left( {y_{i} ,f\left( {x_{i} } \right)} \right) + \lambda\,||f||{^{2}_{K}}$$

where *V* was a smooth loss function, $||f||_{K}$ was the norm of the prediction function *f* associated to the kernel *K*, and λ > 0 was a regularization parameter balancing the prediction error and the complexity of the model. Then considering that we aimed to obtain a function *f*, which could assign close value for every disease–miRNA pairs compared with their initial values in *S*, we could use the following simple square-loss function:

11 $$V\left( {y_{i} ,f\left( {x_{i} } \right)} \right) = (y_{i} - f\left( {x_{i} } \right))^{2}$$

Based on the Representer Theorem [56], the solution of Eq. 9 could be written in the following form:

12 $$f\left( {x_{i} } \right) = \mathop \sum \limits_{i = 1}^{n} \alpha_{i} K\left( {x,x_{i} } \right)$$

Furthermore, with the fact that $||f||_{K}^{2} =\varvec{\alpha}^{T} K\varvec{\alpha}$ [57] we could obtain the classification function for single kernel problem:

13 $$minF\left(\varvec{\alpha}\right) = \mathop {\hbox{min} }\limits_{{\varvec{\alpha}\in R^{n} }} \frac{1}{2n}\mathop \sum \limits_{i = 1}^{n} \left( {\varvec{y} - K\varvec{\alpha}} \right)^{T} \left( {\varvec{y} - K\varvec{\alpha}} \right) + \frac{\lambda }{2}\varvec{\alpha}^{T} K\varvec{\alpha}$$

Hence if $\varvec{\alpha}$ could be calculated, the prediction score for all the disease–miRNA pairs in *S* could be obtained.

In fact, according to previous study [55],**α** could be obtained by solving a single of system linear equations:

14 $$\left( {K + \lambda \varvec{I}} \right)\varvec{\alpha}= \varvec{y}$$

In single kernel situation, we could construct such pairwise kernel *K* as the Kronecker product of the two base kernels [58]: $K = K_{D} \otimes K_{M}$. Unfortunately, the Kronecker product kernel directly would involve calculating the inverse of an (*nd* × *nm*) × (*nd* × *nm*) matrix, which would take O((nd × nm)^3^) operations. Thus, the size of the base kernel matrix made the model training computationally unfeasible even for moderate number of diseases and miRNAs. Hence, in order to make training process more efficient, we could further take advantage of two specific algebraic properties of the Kronecker product [59] and use the eigendecomposition of the Kronecker product [60] to calculate $\varvec{\alpha}$.

Let $K_{D} = Q_{D} \varLambda_{D} Q_{D}^{T}$ and $K_{M} = Q_{M} \varLambda_{M} Q_{M}^{T}$ be the eigendecomposition of the kernel matrices $K_{D}$ and $K_{M}$. Since the eigenvalues (vectors) of a Kronecker product are the Kronecker product of eigenvalues (vectors), for Eq. 13, the solution $\varvec{\alpha}$ can be calculated by Kronecker-RLS method as follows [60]:

15 $$\varvec{\alpha}= vec\left( {Q_{M} CQ_{D}^{T} } \right)$$

where *vec*(·) stacked the columns of a matrix into a vector, and *C* was a matrix defined as: $vec\left( C \right) = \left( {\varLambda_{D} \otimes \varLambda_{M} } \right)\left( {\varLambda_{D} \otimes \varLambda_{M} + \lambda I} \right)^{ - 1} vec\left( {Q_{M}^{T} Y^{T} Q_{D} } \right)$.

So far, the single kernel problem had been introduced, and the solution, $\varvec{\alpha }$, could be calculated successfully and efficiently.

Next, we would introduce how MKRMDA could be designed for multiple kernels problem, which meant that MKRMDA could integrate more similarity information about disease and miRNA. It was natural that if we could combine different kernels by an optimized and reasonable way, we could make the best of relevant data information. We considered various base kernels for diseases and miRNAs as $\varvec{K}_{D} = \left( {K_{D}^{1} ,K_{D}^{2} , \ldots ,K_{D}^{{P_{D} }} } \right)\;{\text{and }}\varvec{K}_{M} = \left( {K_{M}^{1} ,K_{M}^{2} , \ldots ,K_{M}^{{P_{M} }} } \right),P_{D}$ and $P_{M}$ were the number of base kernels investigated for diseases and miRNAs, respectively. In MKRMDA, different base kernels could be finally combined by a linear function, such as $K_{D}^{*} \;{\text{and}}\;K_{M}^{*}$:

16 $$K_{D}^{*} = \mathop \sum \limits_{i = 1}^{{P_{D} }} \beta_{D}^{i} K_{D}^{i} , K_{M}^{*} = \mathop \sum \limits_{j = 1}^{{P_{M} }} \beta_{M}^{j} K_{M}^{j}$$

where $\varvec{\beta}_{\varvec{D}} = \left\{ {\beta_{D}^{1} ,\beta_{D}^{2} , \ldots \beta_{D}^{{P_{D} }} } \right\}$ and $\varvec{\beta}_{\varvec{M}} = \left\{ {\beta_{M}^{1} ,\beta_{M}^{2} , \ldots \beta_{M}^{{P_{M} }} } \right\}$ corresponded to the weights of disease and miRNA kernels, respectively. Then $K_{D}^{*} \;{\text{and}}\;K_{M}^{*}$ could be used as single base kernel for disease and miRNA, which suited for single kernel problem. To obtain optimal $\varvec{\beta}_{\varvec{D}} \;{\text{and}}\;\varvec{\beta}_{\varvec{M}}$, we used a two-step optimization process [49], in which the optimization of the vector $\varvec{a}$ was interleaved with the optimization of the kernel weights. Step 1 was that given two initial weight vectors, $\varvec{\beta}_{\varvec{D}}^{0} \;{\text{and}}\;\varvec{\beta}_{\varvec{M}}^{0}$, an optimal value for the vector $\varvec{a}$ could be calculated by Eq. 14. Step 2 was that using the optimized $\varvec{a}$, we could proceed to find optimal $\varvec{\beta}_{\varvec{D}} \;{\text{and}}\;\varvec{\beta}_{\varvec{M}}$. These two steps were repeated until convergence, resulting in the finally optimal $K_{D}^{*} \;{\text{and}}\;K_{M}^{*}$ for disease and miRNA, respectively (due to limited space, for further information, see Additional file 1).

**Fig. 1:**
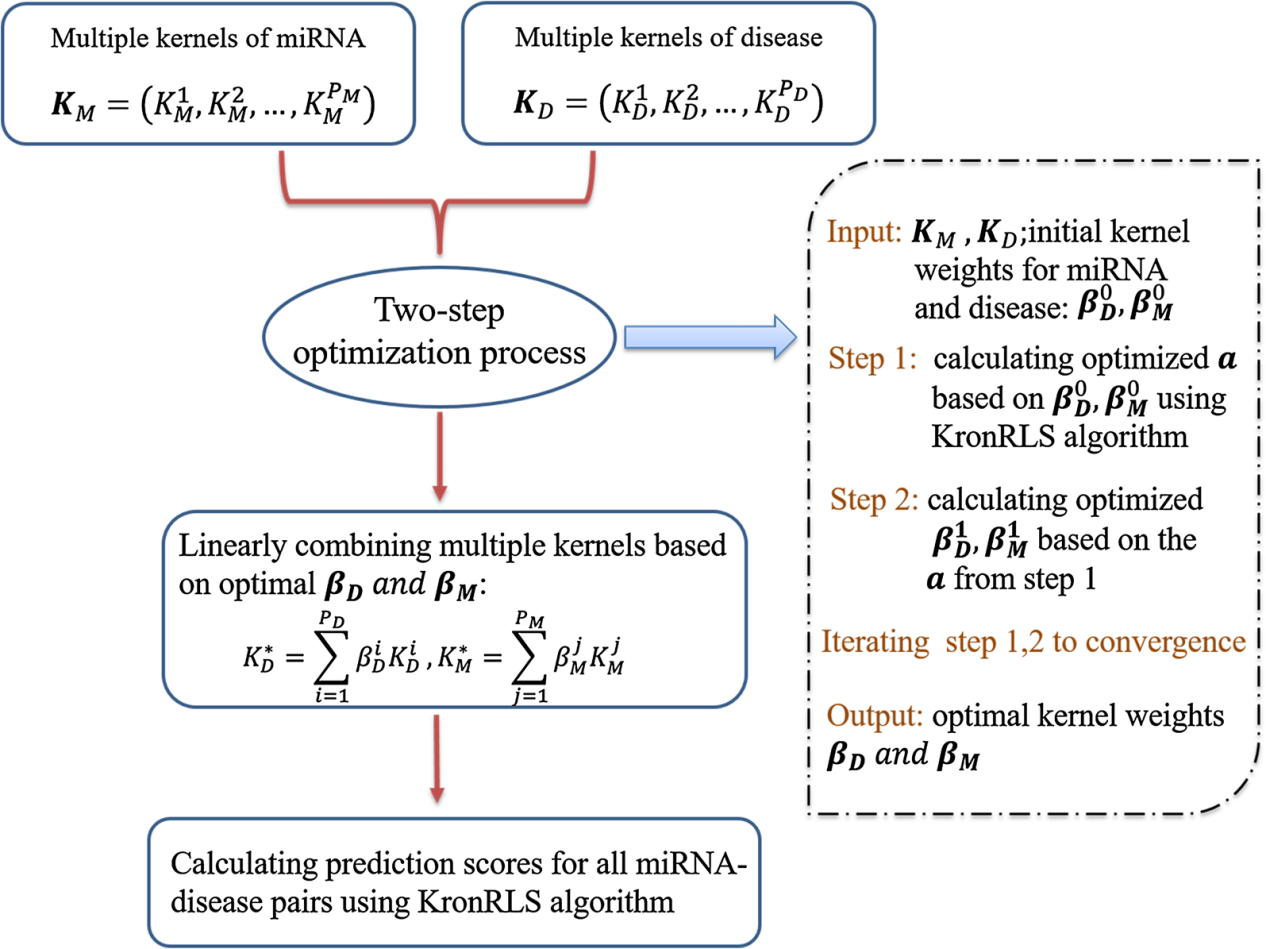
Flowchart of MKRMDA model to predict potential miRNA–disease associations based on multiple kernels of miRNA and disease and known miRNA–disease associations in HMDDv2.0 database

As mentioned before, after this two-step optimization process reached the convergence, we obtained the optimized single kernel both for disease and miRNA, $K_{D}^{*} \;{\text{and}}\;K_{M}^{*}$, then we could make use of these two kernels in single kernel situation introduced before, finally the prediction scores for all disease–miRNAs pairs were generated by MKRMDA (see Fig. 1).

Additionally, in our model, we set the mean of all the base kernels of miRNA and disease as the initial value for the two-step optimization iterative process, which was employed to further calculate the optimal kernel weights for multiple kernels involved as mentioned above. The mean disease kernel was computed as $K_{D}^{*} = 1/P_{D} \mathop \sum \nolimits_{i = 1}^{{P_{D} }} K_{D}^{i}$, and the same could be done for miRNAs, analogously. In addition, the λ parameter was evaluated in the interval $\left\{ {2^{ - 15} ,2^{ - 10} , \ldots ,2^{30} } \right\}$. The σ regularization coefficient was also optimized in the interval $\left\{ {0,0.25,0.5,0.75,1} \right\}$.

## Results

### Cross validation

LOOCV was often implemented to evaluate the performance of prediction model. In this work, we conducted LOOCV in two different ways: global and local LOOCV. Like the meaning of ‘local’, local LOOCV was implemented as follows: firstly, we chose a disease, then each known miRNA associated with this chosen disease was left out in turn as test sample and the other associated miRNAs were used as seed samples, thirdly each time we ranked the predicted association probability of current test sample with the candidate samples, which were the miRNAs without known association with the chosen disease. If the rank of the test miRNA exceeded the given threshold, the model was considered to successfully predict this miRNA–disease association. While, global LOOCV was implemented in a different way: firstly, we considered all the diseases simultaneously, which meant that each time the known disease–miRNA associations in HMDD v2.0 was left out in turn as test sample. Then all the other associations were set as seed samples and all the unknown associations were considered as candidate samples. Thirdly, same as local method, if the rank of test association exceeded the given threshold, the model was considered to successfully predict this association.

Furthermore, receiver-operating characteristics (ROC) curve was drawn by plotting true positive rate (TPR, sensitivity) against false positive rate (FPR, 1-specificity) at different thresholds. Specifically, sensitivity was denoted as the percentage of the correctly identified positive samples among all the positives. Meanwhile, specificity was denoted as the percentage of negative miRNA–disease pairs ranked below the threshold among all negatives. Furthermore, the predictive performance of MKRMDA could be evaluated by calculating the area under ROC curve (AUC). Specifically, AUC = 1 meant the perfect predictive performance of the model, and AUC = 0.5 indicated a random performance.

**Fig. 2:**
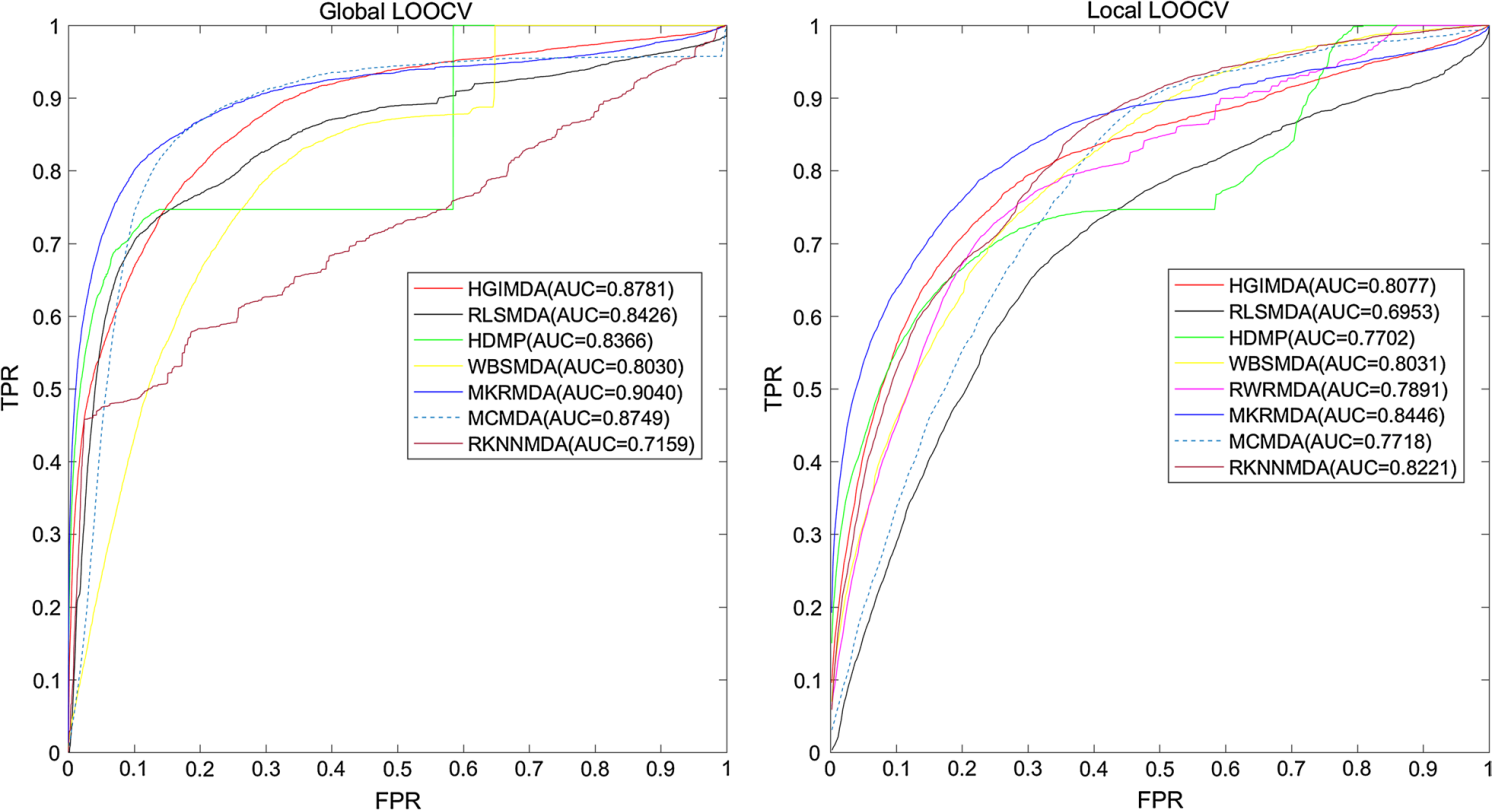
Performance comparisons between MKRMDA and some state-of-the-art disease–miRNA association prediction models (HGIMDA, RLSMDA, HDMP, sWBSMDA, MCMDA and RKNNMDA) in terms of ROC curve and AUC based on local and global LOOCV, respectively. As a result, MKRMDA achieved AUCs of 0.9040 and 0.8446 in the global and local LOOCV, which represents more outstanding prediction performance than all the previous classical models

Figure 2 showed the performance comparisons of the global and local LOOCV results between several computational models. As shown in the figure, MKRMDA, HGIMDA, RLSMDA, HDMP, WBSMDA, MCMDA, RKNNMDA obtained AUCs of 0.9040, 0.8781, 0.8426, 0.8366, 0.8030, 0.8749 and 0.7159 in the global LOOCV, respectively. For the local LOOCV, MKRMDA, HGIMDA, RLSMDA, HDMP, WBSMDA, RWRMDA, MCMDA and RKNNMDA obtained AUCs of 0.8446, 0.8077, 0.6953, 0.7702, 0.8031, 0.7891, 0.7718 and 0.8221, respectively. The MaxFlow model obtained AUC of 0.8693 according to their paper, was also a little lower than MKRMDA’s. RWRMDA model could not implement global LOOCV because this model could not be implemented for all the diseases simultaneously. Additionally, RBMMMDA [48] was not included in the comparison with MKRMDA because the result of RBMMMDA were the corresponding association types between miRNAs and diseases, which were different from the input and output of our algorithm. As a result, MKRMDA had shown excellent and reliable prediction performance. We thought that MKRMDA may provide potential reference value for miRNA–disease association predictive experiments.

In addition, we also adopted fivefold cross validation for prediction evaluation, which was conducted in this way: all the known miRNA–disease associations were randomly divided into 5 groups with equal sizes, then each of the 5 groups was set as test samples and the other groups as training samples. Hence, when a group test samples was chosen, MKRMDA would be implemented and the prediction scores of every test sample in this group would be compared with the scores of candidate miRNAs. To reduce the possible impact caused by random divisions in the process of obtaining test samples, fivefold cross validation was conducted 100 times. Finally, MKRMDA achieved reliable performance with AUC of 0.8894 ± 0.0015, higher than those generated by other models, such as RLSMDA: 0.8569 ± 0.0020; HDMP: 0.8342 ± 0.0010; WBSMDA: 0.8185 ± 0.0009 MCMDA: 0.8767 ± 0.0011; RKNNMDA: 0.6723 ± 0.0027.

### Case studies

MKRMDA had been applied to predict potential miRNA–disease associations for all the diseases investigated in this paper. To further demonstrate the prediction ability of MKRMDA, as mentioned before, three ways of case studies were carried out. Case studies on colonic cancer, esophageal cancer and lymphoma were implemented in the first way of case study, in which the disease–miRNA associations recorded in HMDDv2.0 [52] were used as training samples and miRNAs without known associations with currently considered diseases were regarded as test samples. After MKRMDA was implemented, we verified the top 50 miRNAs predicted to be associated with corresponding disease based on the experimental associations recorded in miR2Disease [61] and dbDEMC database [62].

Colonic cancer is a complex disease in which cancer cells form in the tissues of the colon, and colonic cancer is reported to the second leading cause of cancer death in the United States with the 5 year survival rates of 65% in the United States [63]. As many colonic cancers arise from adenomatous polyps without obvious symptoms, screening test for this cancer is effective not only for early detection but also for prevention. Additionally, with the rapid development of high-throughput sequencing technologies, researchers have identified many miRNAs associated with colonic cancer. For example, miR-141 and miR-200b were confirmed to be highly overexpressed in colonic cancer [64]. In the case study for colonic cancer, candidate miRNAs were prioritized according to the scores obtained from MKRMDA, as a result, 38 out of top 50 were confirmed by recent experimental results in miR2Disease and dbDEMC (see Table 1). For example, miR-183, highly ranked and confirmed by miR2Disease and dbDEMC databases simultaneously, was significantly deregulated in colorectal cancer cells [65].

**Table 1:** We implemented MKRMDA on colonic cancer for potential disease–related miRNA prediction and conducted the first pattern of case study, in which the disease–miRNA associations recorded in HMDDv2.0 were used as training samples and miRNAs without known associations with currently considered diseases were regarded as test samples

miRNA	Evidence	miRNA	Evidence
hsa-mir-222	dbdemc	hsa-mir-199b	dbdemc
hsa-mir-150	Unconfirmed	hsa-mir-30d	dbdemc
hsa-mir-146b	Unconfirmed	hsa-mir-130a	Unconfirmed
hsa-mir-200a	Unconfirmed	hsa-mir-375	Unconfirmed
hsa-mir-199a	Unconfirmed	hsa-mir-194	dbdemc, miR2Disease
hsa-mir-183	dbdemc; miR2Disease	hsa-mir-18b	Unconfirmed
hsa-mir-196a	dbdemc; miR2Disease	hsa-mir-27a	miR2Disease
hsa-mir-203	dbdemc; miR2Disease	hsa-mir-93	dbdemc; miR2Disease
hsa-mir-181b	dbdemc; miR2Disease	hsa-mir-7	dbdemc; miR2Disease
hsa-mir-210	dbdemc	hsa-mir-373	dbdemc
hsa-mir-135b	dbdemc; miR2Disease	hsa-mir-98	Unconfirmed
hsa-mir-34c	miR2Disease	hsa-mir-124	dbdemc
hsa-mir-135a	dbdemc	hsa-mir-30b	dbdemc
hsa-mir-148a	dbdemc	hsa-mir-339	dbdemc; miR2Disease
hsa-mir-29c	dbdemc	hsa-mir-95	dbdemc; miR2Disease
hsa-mir-195	dbdemc; miR2Disease	hsa-mir-30e	Unconfirmed
hsa-mir-34b	dbdemc; miR2Disease	hsa-mir-302c	Unconfirmed
hsa-mir-92b	Unconfirmed	hsa-mir-27b	dbdemc; miR2Disease
hsa-mir-181a	dbdemc; miR2Disease	hsa-mir-206	dbdemc
hsa-mir-133a	dbdemc; miR2Disease	hsa-mir-99a	dbdemc
hsa-mir-25	dbdemc; miR2Disease	hsa-mir-451	miR2Disease
hsa-mir-26a	dbdemc; miR2Disease	hsa-mir-182	dbdemc; miR2Disease
hsa-mir-214	dbdemc	hsa-mir-224	dbdemc; miR2Disease
hsa-mir-15b	miR2Disease	hsa-mir-20b	Unconfirmed
hsa-mir-429	dbdemc	hsa-mir-219	dbdemc

According to the prediction results, among the top 10 and 50 potential colonic cancer related miRNAs, 6 and 38 were confirmed by miR2Disease and dbDEMC databases

Esophageal cancer is the eighth common cancer worldwide and is one of the deadliest cancers worldwide because of its extremely aggressive nature and poor survival rate [66]. The overall 5-year survival of esophageal cancer ranges from 15 to 25% [67, 68]. There is research suggesting that the survival rate could increase to 90% if the tumors could be diagnosed at an early stage [69]. Therefore, the early detection is vital for timely treatment of esophageal cancers [70]. Many miRNAs have been reported to be related with esophageal cancers. For example, by post-transcriptionally regulating enhancer of zestehomolog 2, miR-214 and miR-98 could suppress migration and invasion in human esophageal squamous cell carcinoma [71]. As mentioned before, in the first way of case study for esophageal cancer, 47 out of top 50 predicted miRNAs for esophageal cancer were confirmed by at least one of miR2Disease and dbDEMC databases (see Table 2).

**Table 2:** We implemented MKRMDA on esophageal cancer for potential disease–related miRNA prediction and conducted the first pattern of case study according to the prediction results

miRNA	Evidence	miRNA	Evidence
hsa-mir-200b	dbdemc	hsa-mir-18b	dbdemc
hsa-mir-1	dbdemc	hsa-mir-29a	Unconfirmed
hsa-mir-125b	dbdemc	hsa-let-7f	dbdemc
hsa-mir-142	dbdemc	hsa-mir-146b	dbdemc
hsa-mir-18a	dbdemc	hsa-mir-7	dbdemc
hsa-mir-16	dbdemc	hsa-mir-497	dbdemc
hsa-mir-17	dbdemc	hsa-mir-191	dbdemc
hsa-let-7e	dbdemc	hsa-mir-106a	dbdemc
hsa-mir-429	dbdemc	hsa-mir-132	dbdemc
hsa-mir-222	dbdemc	hsa-let-7g	dbdemc
hsa-mir-218	Unconfirmed	hsa-mir-9	dbdemc
hsa-mir-221	dbdemc	hsa-mir-182	dbdemc
hsa-mir-199b	dbdemc	hsa-mir-122	Unconfirmed
hsa-mir-133b	dbdemc	hsa-mir-424	dbdemc
hsa-mir-125a	dbdemc	hsa-mir-24	dbdemc
hsa-mir-19b	dbdemc	hsa-mir-181a	dbdemc
hsa-mir-195	dbdemc	hsa-mir-224	dbdemc
hsa-let-7i	dbdemc	hsa-mir-335	dbdemc
hsa-mir-107	dbdemc; miR2Disease	hsa-mir-181b	dbdemc
hsa-mir-194	dbdemc; miR2Disease	hsa-mir-302b	dbdemc
hsa-mir-30a	dbdemc	hsa-mir-151	dbdemc
hsa-let-7d	dbdemc	hsa-mir-302c	dbdemc
hsa-mir-30c	dbdemc	hsa-mir-372	dbdemc
hsa-mir-127	dbdemc	hsa-mir-491	dbdemc
hsa-mir-10b	dbdemc	hsa-mir-32	dbdemc

As a result, among the top 10 and 50 potential esophageal cancer related miRNAs, 10 and 47 were confirmed by miR2Disease and dbDEMC databases

Lymphoma is a group of blood cell tumors that develop from lymphocytes and lymphoma most often spreads to the lungs, liver, and brain. The two main types of lymphoma are Hodgkin lymphoma and non-Hodgkin lymphoma (NHL) [72]. Meanwhile, lymphomas, including HL and NHL, are reported as the seventh-most lethality cancers worldwide and lymphomas are also the third-most common cancer in children [72]. However, lymphomas may be curable if detected in early stages with modern treatment. Recent experimental research found that miR-17-5p showed an increased expression level compared with normal canine peripheral blood mononuclear cells and normal lymph nodes (LN). In the case study on lymphoma, for the top 50 predicted lymphoma-associated miRNAs ranked by MKRMDA, we had 44 associations confirmed by experimental literature evidences (see Table 3).

**Table 3:** We also implemented MKRMDA for potential lymphoma-related miRNA prediction and conducted the first pattern of case study based on the prediction results

miRNA	Evidence	miRNA	Evidence
hsa-mir-125b	Unconfirmed	hsa-mir-22	dbdemc
hsa-mir-223	dbdemc	hsa-let-7b	dbdemc
hsa-mir-34a	dbdemc	hsa-mir-199b	dbdemc
hsa-mir-9	dbdemc	hsa-mir-494	dbdemc
hsa-mir-221	dbdemc, miR2Disease	hsa-mir-10a	dbdemc; miR2Disease
hsa-mir-195	dbdemc	hsa-mir-26b	dbdemc
hsa-mir-145	dbdemc, miR2Disease	hsa-mir-100	dbdemc
hsa-let-7a	dbdemc	hsa-mir-27a	dbdemc
hsa-mir-224	dbdemc	hsa-mir-30e	dbdemc
hsa-mir-183	dbdemc	hsa-mir-127	dbdemc; miR2Disease
hsa-mir-142	Unconfirmed	hsa-mir-137	dbdemc
hsa-mir-29a	dbdemc	hsa-mir-1	dbdemc
hsa-mir-31	dbdemc	hsa-mir-34c	Unconfirmed
hsa-mir-106a	dbdemc; miR2Disease	hsa-mir-34b	dbdemc
hsa-mir-181b	dbdemc	hsa-mir-192	dbdemc
hsa-mir-143	dbdemc; miR2Disease	hsa-mir-196b	Unconfirmed
hsa-mir-205	dbdemc	hsa-mir-199a	dbdemc
hsa-mir-182	dbdemc	hsa-mir-129	dbdemc
hsa-mir-29b	dbdemc	hsa-mir-99b	dbdemc
hsa-mir-222	dbdemc	hsa-mir-30a	dbdemc
hsa-mir-96	dbdemc	hsa-let-7d	dbdemc
hsa-mir-10b	dbdemc	hsa-mir-23b	dbdemc
hsa-mir-106b	dbdemc	hsa-mir-148a	dbdemc
hsa-mir-542	Unconfirmed	hsa-mir-429	Unconfirmed
hsa-mir-141	dbdemc	hsa-mir-27b	dbdemc

As a result, among the top 10 and 50 potential lymphoma-related miRNAs, 9 and 44 were confirmed by miR2Disease and dbDEMC databases

These 3 cancers were chosen mainly because these 3 cancers (included some other cancers) were very important and these cancers were often taken as case studies in many computational models such as HGIMDA (colonic cancer, esophageal cancer), RKNNMDA (colonic cancer, esophageal cancer), MCMDA (colonic cancer, lymphoma) and so on. What’s more, we also compared the confirmed case studies results of HGIMDA and RKNNMDA on previously mentioned three cancers for the top 50 predicted miRNAs (see Additional file 1). We chose these two models because they were ranked first in the models whose performance were compared with our computational model in the global LOOCV and local LOOCV, respectively.

In addition, we conducted case study of hepatocellular carcinoma (HCC) in the second way, in which we removed all the related miRNAs information of HCC to model the situation where a new disease without known miRNA associations was investigated. Then we verified the prediction results of HCC with HMDD v2.0 database, miR2Disease, and dbDEMC database. Hepatocellular carcinoma is the most common type of liver cancer. Meanwhile, HCC is the sixth most prevalent cancer and the third most frequent cause of cancer-related death [73]. More than 30 miRNAs have been validated to be related to the development of HCC in the gold standard dataset. For example, the expression of miR-125a and miR-99b were quite lower in HCC compared to normal liver [74]. MiR-122a was a liver-specific miRNA and it was frequently downregulated in HCC [75]. Among the top 50 predicted potential HCC-related miRNAs, there were 44 miRNAs confirmed by aforementioned various databases, i.e. HMDDv2.0, miR2Disease and dbDEMC database (see Table 4). For example, miR-21, which was ranked first in the top 50 predicted miRNAs, had been reported to be up-regulated in patients with HCC and it had strong potential to serve as novel biomarker for liver injury [76].

**Table 4:** We conducted case study of hepatocellular carcinoma in the second way, in which we removed all the hepatocellular carcinoma related miRNAs information to simulate a new disease without any known associations

miRNA	Evidence	miRNA	Evidence
hsa-mir-21	HMDDv2	hsa-mir-571	Unconfirmed
hsa-mir-122	dbdemc; HMDDv2	hsa-mir-133b	HMDDv2
hsa-mir-375	HMDDv2	hsa-mir-34a	dbdemc; HMDDv2
hsa-mir-145	dbdemc; HMDDv2	hsa-let-7b	HMDDv2
hsa-mir-200c	HMDDv2	hsa-mir-138	HMDDv2
hsa-mir-200b	HMDDv2	hsa-mir-100	dbdemc; HMDDv2
hsa-mir-200a	dbdemc; HMDDv2	hsa-mir-148a	dbdemc; HMDDv2
hsa-mir-451a	HMDDv2	hsa-mir-26b	dbDEMC
hsa-mir-124	HMDDv2	hsa-mir-214	dbdemc; HMDDv2
hsa-mir-486	HMDDv2	hsa-mir-199a	dbdemc; HMDDv2
hsa-mir-210	dbdemc; HMDDv2	hsa-mir-625	Unconfirmed
hsa-mir-16	dbdemc; HMDDv2	hsa-mir-370	HMDDv2
hsa-mir-10b	HMDDv2	hsa-mir-23a	dbdemc; HMDDv2
hsa-mir-629	HMDDv2	hsa-mir-708	Unconfirmed
hsa-mir-126	dbdemc; HMDDv2	hsa-mir-499a	HMDDv2
hsa-mir-196a	HMDDv2	hsa-mir-184	Unconfirmed
hsa-mir-31	HMDDv2	hsa-mir-378a	HMDDv2
hsa-mir-425	HMDDv2	hsa-mir-141	HMDDv2
hsa-mir-143	dbdemc	hsa-mir-548d	Unconfirmed
hsa-mir-182	HMDDv2	hsa-mir-25	dbdemc; HMDDv2
hsa-let-7i	dbdemc; HMDDv2	hsa-mir-34c	HMDDv2
hsa-mir-222	dbdemc; HMDDv2	hsa-mir-1290	HMDDv2
hsa-mir-26a	dbdemc; HMDDv2	hsa-mir-494	Unconfirmed
hsa-mir-155	dbdemc; HMDDv2	hsa-mir-320b	HMDDv2
hsa-let-7a	dbdemc; HMDDv2	hsa-mir-105	HMDDv2

Then we verified the prediction results based on HMDD v2.0 database, miR2Disease, and dbDEMC database. As a result, among the top 10 and 50 potential miRNAs, 10 and 44 were confirmed

Furthermore, to test the robustness of MKRMDA, we presented case study for breast cancer in the third way, in which we only used the known disease–miRNA associations in HMDDv1.0 database as training samples and used associations in HMDD v2.0 database, miR2Disease, and dbDEMC database as test datasets. Breast cancer is currently reported as the deadliest cancer in women, accounting for 25% of all cancer caused death cases [72]. Specifically, breast cancer is more common in developed countries and is about 100 times more common in women than in men. The majority deaths of the breast cancer come from the developing countries, where most of the women are diagnosed in late stages [77]. There are about 176 miRNAs known to be related to the breast cancer in the golden standard dataset. For example, miR-122 was down-regulated in breast cancer cells, while, the expression levels of miR-10b and miR-21 were reported significantly increased in the CSF (cerebrospinal fluid) of patients with breast cancer, compared with patients in nonneoplastic conditions [78, 79]. We implemented MKRMDA to prioritize candidate miRNAs without the known associations with breast cancer in HMDDv1.0. As a result, among the top 50 potential breast cancer-related miRNAs, there were 47 associations which have been verified by known miRNA–disease associations in at least one of HMDD v2.0 database, miR2Disease, and dbDEMC database (see Table 5).

**Table 5:** We presented a case study for breast cancer in the third way of case study, in which we only used known disease–miRNA association based on HMDDv1.0 database as test samples to assess the robustness of the prediction model, and then we verified the prediction results according to the experimental confirmed disease–miRNA associations recorded in HMDD v2.0 database, miR2Disease, and dbDEMC database

miRNA	Evidence	miRNA	Evidence
hsa-let-7b	dbdemc; HMDDv2	hsa-mir-130b	dbdemc
hsa-let-7e	dbdemc; HMDDv2	hsa-mir-363	dbdemc
hsa-mir-223	dbdemc; HMDDv2	hsa-mir-27a	dbdemc; miR2Disease; HMDDv2
hsa-mir-191	dbdemc; miR2Disease; HMDDv2	hsa-mir-198	dbDEMC
hsa-let-7i	dbdemc; miR2Disease; HMDDv2	hsa-mir-520c	miR2Disease; HMDDv2
hsa-mir-101	dbdemc; miR2Disease; HMDDv2	hsa-mir-521	dbdemc
hsa-mir-92a	HMDDv2	hsa-mir-520b	dbdemc; HMDDv2
hsa-let-7g	dbdemc; HMDDv2	hsa-mir-95	dbdemc
hsa-let-7c	dbdemc; HMDDv2	hsa-mir-128b	miR2Disease
hsa-mir-92b	dbdemc	hsa-mir-142	Unconfirmed
hsa-mir-16	dbdemc; HMDDv2	hsa-mir-15b	dbdemc
hsa-mir-106a	dbdemc	hsa-mir-100	dbdemc; HMDDv2
hsa-mir-32	dbdemc	hsa-mir-30e	Unconfirmed
hsa-mir-203	dbdemc; miR2Disease; HMDDv2	hsa-mir-491	dbdemc
hsa-mir-126	dbdemc; miR2Disease; HMDDv2	hsa-mir-182	dbdemc; miR2Disease; HMDDv2
hsa-mir-373	dbdemc; miR2Disease; HMDDv2	hsa-mir-130a	dbDEMC
hsa-mir-99a	dbDEMC	hsa-mir-199b	dbdemc; miR2Disease; HMDDv2
hsa-mir-532	dbDEMC	hsa-mir-184	dbdemc
hsa-mir-18b	dbdemc; HMDDv2	hsa-mir-455	dbdemc
hsa-mir-335	dbdemc; miR2Disease; HMDDv2	hsa-mir-139	dbdemc; HMDDv2
hsa-mir-24	dbdemc; HMDDv2	hsa-mir-107	dbdemc; HMDDv2
hsa-mir-181a	dbdemc; miR2Disease; HMDDv2	hsa-mir-186	dbdemc
hsa-mir-124	dbdemc; HMDDv2	hsa-mir-99b	dbdemc
hsa-mir-30a	miR2Disease; HMDDv2	hsa-mir-29c	dbdemc; miR2Disease; HMDDv2
hsa-mir-196b	dbdemc	hsa-mir-542	Unconfirmed

As a result, among the top 10 and 50 potential breast cancer related miRNAs, 10 and 47 were confirmed

In conclusion, the promising results obtained from LOOCV, fivefold cross validation and case studies in three different ways had demonstrated the reliable prediction performance of MKRMDA. Therefore, we further prioritized all the candidate miRNAs for all the diseases recorded in HMDD v2.0 database. The predicted ranks of miRNAs for each disease were publicly released for further experimental validation (see Additional file 2). A higher prediction score meant a higher association probability of the corresponding disease and miRNA. While, we had to point out that the negative scores did not mean that the relevant miRNA and disease were negatively correlated. Our case studies focus on the top prediction scores, which generally were all positive. The potential disease–miRNA associations with relatively high ranks were expected to be confirmed by biological experiments and clinical observation in the future.

## Discussion

The excellent and reliable prediction performance of MKRMDA could largely be owed to the following several factors. Firstly, the known experimentally confirmed disease–miRNA associations in HMDDv2.0, which we used as training samples in the prediction process, were abundant and reliable. Secondly, MKRMDA fully took advantage of heterogeneous datasets (known disease–miRNA associations, miRNA functional similarity, disease semantic similarity, Gaussian interaction profile kernel similarity for miRNAs and diseases) to predict the potential associations. Thirdly, MKRMDA used a two-step optimization process to automatically optimize the combination of the involved multiple kernels in the prediction progress, which significantly improved the prediction performance. Additionally, MKRMDA conquered the memory limitation difficulty by using some algebraic properties of Kronecker product. All in all, MKRMDA could handle data from different resources by two-step optimal decision for automatically combining them to fully take use of them for biology research or multisource data fusion research.

Of course, MKRMDA also needs to be improved in the future for the reasons as follows: first, MKRMDA was developed mainly based on the assumption that functionally similar miRNAs were more likely to have associations with phenotypically similar diseases, which might cause bias to miRNAs with more known associated diseases. Furthermore, how to appropriately choose proper values for the parameters involved in the model of MKRMDA from the alternative values need to be further solved. In addition, in the optimization iterative procedure, the method used to set initial values might also be opportunely improved to get more reliable prediction result.

## Conclusion

Identifying novel miRNA–disease associations is a vitally important goal of biological development, and it also plays a critical role in the understanding of disease pathogenesis at the miRNA level. In this paper, we proposed the computational method, MKRMDA, to predict potential diseases related miRNAs. The performance of MKRMDA was evaluated by implementing LOOCV and fivefold cross validation based on the known experimentally verified miRNA–disease associations. The AUC scores, 0.9040 in global LOOCV and 0.8446 in local LOOCV, demonstrated the reliable and effective performance of MKRMDA. Moreover, we implemented three different kinds of case studies for further evaluations. As mentioned before, in the first case study, 38, 47, and 44 out of top 50 predicted miRNAs for colonic cancer, esophageal cancer, and lymphoma were verified by recent experimental reports, respectively. In the second and third way of case study for hepatocellular carcinoma and breast cancer, 44 and 47 out of top 50 predicted miRNAs were verified by recent experimental researches, respectively. All of these showed the reliable performance of MKRMDA. It was anticipated that MKRMDA could be an important and valuable computational tool for miRNA–disease association prediction and miRNA biomarker identification for human disease diagnosis, treatment, prognosis and prevention. In addition, MKRMDA was well suited for research situations where abundant kernel-related data from different resources was provided, especially when researchers expected to find an appropriate and optimal method to combine the different types of relevant data for the best use of them. All the above-mentioned results sufficiently showed the reliability of MKRMDA in predicting potential disease–miRNA associations. MKRMDA was hoped to be helpful for miRNA–disease association prediction and relevant miRNA research from the perspective of computational biology.

## Additional files

**Additional file 1.** Additional information about the multiple kernel learning method, two-step optimization process and the case studies comparison with HGIMDA and RKNNMDA.

**Additional file 2.** We further applied MKRMDA to predict candidate miRNAs for all the diseases involved in HMDDv2.0. Prediction results were publicly released for further research and experimental validation.

**Additional file 3.** The file of codes and data used in MKRMDA.

## Abbreviations

MiRNA: microRNA
LOOCV: leave-one-out cross validation
fivefold CV: fivefold cross validation
ROC: receiver-operating characteristics curve
AUC: the area under ROC curve
