# Supplementary material for: MKRMDA: multiple kernel learning-based Kronecker regularized least squares for MiRNA–disease association prediction
Source: J Transl Med. 2017 Dec 12;15:251. doi: 10.1186/s12967-017-1340-3 (PMC5727873; doi:10.1186/s12967-017-1340-3)
Supplement: Supplementary file 1 — Additional file 1. Additional information about the multiple kernel learning method, two-step optimization process and the case studies comparison with HGIMDA and RKNNMDA. [file 12967_2017_1340_MOESM1_ESM.docx]

| Research article  **MKRMDA: Multiple kernel learning-based Kronecker Regularized least squares for MiRNA-Disease Association prediction**  Xing Chen ^1,*^, Ya-Wei Niu ^2^, Guang-Hui Wang ^2,*^, Gui-Ying Yan ^3^  ^1^School of Information and Control Engineering, China University of Mining and Technology, Xuzhou, 221116, China  ^2^School of mathematics, Shandong University, Jinan, 250100, China  ^3^Academy of Mathematics and Systems Science, Chinese Academy of Sciences, Beijing, 100190, China  **XC:** [**xingchen@cumt.edu.cn**](mailto:xingchen@cumt.edu.cn)**;**  **YN: ywniu3344@126.com;**  **GW: ghwang@sdu.edu.cn;**  **GY: yangy@amss.ac.cn;**  Received on XXXXX; revised on XXXXX; accepted on XXXXX  Associate Editor: XXXXXXX |
| --- |

**Supplementary Information**

1. **MKRMDA METHOD**

In this work, we proposed the MKRMDA model, an extension of the Kron-RLS algorithm, to predict potential diseases related miRNAs involving in multiple kernels. Before introducing our model, we would describe the RLS and the KronRLS algorithms [[1](#_ENREF_1), [2](#_ENREF_2)].

**RLS and KronRLS**

Given a set of diseases $D=\left\{ d_{1},d_{2}\ldots,d_{n_{d}} \right\}$, miRNAs $R=\left\{ r_{1},r_{2}\ldots,r_{n_{r}} \right\}$, and the set of training inputs $x_{i}$ (disease-miRNA pairs) and their binary labels $y_{i}\in R$ (where 1 stands for a known association and 0 otherwise), with $1<i\leq n,nd\times nm$(number of disease-miRNA pairs). The RLS approach minimizes the following function [[3](#_ENREF_3)]:

$J\left( f \right)=\frac{1}{2n}\sum_{i=1}^{n} {(y_{i}-f\left( x_{i} \right))}^{2}+\frac{\lambda}{2}\left\| f \right\|_{K}^{2}$ (1)

where$\left\| f \right\|_{K}$ is the norm of the prediction function *f* on the Hilbert space associated to the kernel K, and λ > 0 is a regularization parameter balancing the prediction error and the complexity of the model. Based on the representer theorem [[4](#_ENREF_4)], a dual representation of the above objective function can be adopted as the following form:

$f\left( x_{i} \right)=\sum_{i=1}^{n} \alpha_{i}K\left( x,x_{i} \right)$(2)

where K: |D||T| × |D||T| → R is the pairwise kernel function and $\boldsymbol{\alpha}$ is the vector of dual variables corresponding to each separation constraint. The RLS algorithm obtains the minimizer of Eq. 1 by solving a system of linear equations $\left( K+\lambda I \right)\boldsymbol{\alpha}=\boldsymbol{y}$, where $\boldsymbol{\alpha}$ and $\boldsymbol{y}$ are both n-dimensional vectors consisting of the parameters $\alpha_{i}$and labels $y_{i}$.

What is more, one can construct such pairwise kernel as the product of two base kernels, namely $K\left( \left( d, r \right), \left( d^{'}, r^{'} \right) \right)= K_{D}(d,d^{'}) K_{R}(r,r^{'})$,where $K_{D}$ and $K_{R}$ are the base kernels for diseases and miRNAs, respectively. This is equivalent to the Kronecker product of the two base kernels [[5](#_ENREF_5), [6](#_ENREF_6)]: $K = K_{D} \otimes K_{R}$. Obviously, the size of the kernel matrix makes the model training computationally unfeasible even for moderate number of diseases and miRNAs.

Furthermore, the KronRLS algorithm is a modification of RLS, it takes advantage of two specific algebraic properties of the Kronecker product to speed up model training [[5](#_ENREF_5)] and uses the eigendecomposition of the Kronecker product [[7](#_ENREF_7), [8](#_ENREF_8)].

Let $K_{D}=Q_{D}\Lambda_{D}Q_{D}^{T}$ and $K_{R}=Q_{R}\Lambda_{R}Q_{R}^{T}$be the eigendecomposition of the kernel matrices $K_{D}$ and $K_{R}$. The solution $\boldsymbol{\alpha}$ can be calculated as follows [[7](#_ENREF_7)]:

$\boldsymbol{\alpha}=vec\left( Q_{R}CQ_{D}^{T} \right)$(3)

where *vec*($\cdot$) is the vectorization operator that stacks the columns of a matrix into a vector, and C is a matrix defined as:

$vec(C)={\left( \Lambda_{D}\otimes\Lambda_{R} \right)\left( \Lambda_{D}\otimes\Lambda_{R}+\lambda I \right)}^{-1}vec\left( Q_{R}^{T}Y^{T}Q_{D} \right)$(4)

Since the estimation of vector $\boldsymbol{\alpha}$ using Eqs. 3 and 4 is much faster than the original RLS estimation process, the KronRLS algorithm is well suited for the large pairwise space involved on the disease-miRNA prediction problem, in such scenario. However, it is not suitable for multiple kernels problem.

**MKRMDA**

In this work, a vector form of different kernels is considered, i.e., $\boldsymbol{K}_{D}=\left( K_{D}^{1},K_{D}^{2},\ldots,K_{D}^{P_{D}} \right) and \boldsymbol{K}_{R}=\left( K_{R}^{1},K_{R}^{2},\ldots,K_{R}^{P_{R}} \right)$, $P_{D}$ and $P_{R}$ indicate the number of base kernels defined over the diseases and miRNAs set, respectively. In this section, we propose an extension of KronRLS to handle multiple kernels.

The different kernels can be combined by a linear function, i.e., the weighted sum of base kernels, corresponding to the optimal kernels $K_{D}^{*}$ and $K_{R}^{*}$:

$K_{D}^{*}=\sum_{i=1}^{P_{D}} \beta_{D}^{i}K_{D}^{i}, K_{R}^{*}=\sum_{j=1}^{P_{R}} \beta_{R}^{j}K_{R}^{j}$ (5)

where $\beta_{D}=\left\{ \beta_{D}^{1},\beta_{D}^{2},\ldots\beta_{D}^{P_{D}} \right\} and \beta_{R}=\left\{ \beta_{R}^{1},\beta_{R}^{2},\ldots\beta_{R}^{P_{R}} \right\}$ correspond to the weights of disease and miRNA kernels, respectively.

The classification function of Eq. 2 can be written in following form, $f_{\alpha}=K\boldsymbol{\alpha}$[[3](#_ENREF_3)] and by applying the well-known property of the Kronecker product, $\left( A\otimes B \right)vec\left( X \right)=vec(BXA^{T})$[[8](#_ENREF_8)], we have:

$f_{\alpha}\left( X \right)=K\boldsymbol{\alpha=}K_{D}^{*}\otimes K_{R}^{*}vec(Q_{R}CQ_{D}^{T})=(K_{R}^{*}(Q_{R}CQ_{D}^{T}){(K_{D}^{*})}^{T})$ (6)

This way, we can rewrite the classification function as $K_{R}^{*}A\left( K_{D}^{*} \right)^{T}$, where $A = unvec(\boldsymbol{a})$. Using the same iterative approach considered in previous MKL researches [[9](#_ENREF_9)], we propose the use of a two-step optimization process, in which the optimization of the vector $\boldsymbol{a}$ is interleaved with the optimization of the kernel weights. Given two initial weight vectors, $\boldsymbol{\beta}_{\boldsymbol{D}}^{\boldsymbol{0}} and \boldsymbol{\beta}_{\boldsymbol{R}}^{\boldsymbol{0}},$ an optimal value for the vector $\boldsymbol{a}$ can be calculated by Eq. 3, and using the optimal $\boldsymbol{a}$, we can proceed to find optimal $\beta_{D} and \beta_{R}$. More specifically, Eq. 1 can be redefined when $\boldsymbol{a}$ is fixed, and knowing that $\left\| f \right\|_{K}^{2}=\boldsymbol{\alpha}^{T}K\boldsymbol{\alpha}$ [[10](#_ENREF_10)], we have: $\boldsymbol{u}=\left( \boldsymbol{y}-\frac{\lambda\boldsymbol{\alpha}}{2} \right)$ (7) Then,

$J\left( f_{\alpha} \right)=\frac{1}{2\lambda n}\left\| \boldsymbol{u}-K\boldsymbol{\alpha} \right\|_{2}^{2}+\frac{1}{2}\boldsymbol{\alpha}^{T}(\boldsymbol{y}-\lambda\boldsymbol{\alpha})$ (8)

Since the second term does not depend on *K*, when $\boldsymbol{y}$ and$\boldsymbol{\alpha}$ are fixed, it can be discarded from the weights optimization procedure. Then we introduce a L2 regularization term, which is parameterized by the σ regularization coefficient, to control sparsity [[11](#_ENREF_11)] of the kernel weights. Additionally, we can convert $\boldsymbol{u}$ to its matrix form by the application of the *unvec* operator, i.e., $U$ = *unvec*($\boldsymbol{u}$), and also use a more appropriate matrix norm (Frobenius, $\left\| A \right\|_{2}\leq\left\| A \right\|_{F}$) [[8](#_ENREF_8)]. Then for any fixed values of $\text{α}$ and $\boldsymbol{\beta}_{\boldsymbol{R}}$, the optimal value for the combination vector can be calculated by solving the optimization problem defined as:

$\min_{\boldsymbol{\beta}_{D}} \frac{1}{2\lambda n}\left\| U-\boldsymbol{m}_{D}\boldsymbol{\beta}_{D} \right\|_{F}+\sigma\left\| \boldsymbol{\beta}_{D} \right\|_{2}^{2}$ (9)

$\boldsymbol{m}_{D}=(K_{R}^{*}A\left( K_{D}^{1} \right)^{T},K_{R}^{*}A\left( K_{D}^{2} \right)^{T},\ldots,K_{R}^{*}A\left( K_{D}^{P_{D}} \right)^{T})$ (10)

while the optimal $\boldsymbol{\beta}_{R}$ can be found fixing the values of $\boldsymbol{\alpha}$ and$\boldsymbol{\beta}_{D}$, according to:

$\min_{\boldsymbol{\beta}_{R}} \frac{1}{2\lambda n}\left\| U-\boldsymbol{\beta}_{R}\boldsymbol{m}_{R} \right\|_{F}+\sigma\left\| \boldsymbol{\beta}_{R} \right\|_{2}^{2}$(11)

$\boldsymbol{m}_{R}=(K_{R}^{1}A\left( K_{D}^{*} \right)^{T},K_{R}^{2}A\left( K_{D}^{*} \right)^{T},\ldots,K_{R}^{P_{R}}A\left( K_{D}^{*} \right)^{T})$ (12)

Additionally, in our work, we considered a simple method available for kernel combination: the mean of base kernels. The mean disease kernel was computed as $K_{D}^{*}=1/{P_{D}\sum_{i=1}^{P_{D}} K_{D}^{i}}$, and the same could be done for miRNAs, analogously. In addition, for the KronRLS-based methods, the λ parameter was evaluated in the interval $\left\{ 2^{-15},2^{-10},\ldots,2^{30} \right\}$. The σ regularization coefficient was also optimized in the interval $\left\{ 0,0.25,0.5,0.75,1 \right\}$.

1. **Case studies comparison with HGIMDA and RKNNMDA**

We compared the confirmed case studies results of HGIMDA and RKNNMDA on previously mentioned three cancers for the top 50 predicted miRNAs. We chose these two models because they were ranked first in the models whose performance were compared with our computational model in the global LOOCV and local LOOCV, respectively.

|  | **MKRMDA** | **HGIMDA** | **RKNNMDA** |
| --- | --- | --- | --- |
| **Colonic cancer** | **38** | **37** | **28** |
| **Esophageal cancer** | **47** | **44** | **40** |
| **Lymphoma** | **44** | **45** | **19** |

**References**

1. van Laarhoven T, Nabuurs SB, Marchiori E: **Gaussian interaction profile kernels for predicting drug-target interaction.** *Bioinformatics* 2011, **27:**3036-3043.

2. Pahikkala T, Airola A, Stock M, Baets BD, Waegeman W: **Efficient regularized least-squares algorithms for conditional ranking on relational data.** *Machine Learning* 2013, **93:**321-356.

3. Rifkin R, Yeo G, Poggio T: **Regularized Least-Squares Classification.** *Acta Electronica Sinica* 2003, **190:**93-104.

4. Kimeldorf G, Wahba G: **Some results on Tchebycheffian spline functions ☆.** *Journal of Mathematical Analysis & Applications* 1971, **33:**82-95.

5. Kashima H, Oyama S, Yamanishi Y, Tsuda K: **On Pairwise Kernels: An Efficient Alternative and Generalization Analysis.** In *Pacific-Asia Conference on Advances in Knowledge Discovery and Data Mining*. 2009: 1030-1037.

6. Yamanishi Y: **Chemogenomic approaches to infer drug-target interaction networks.** *Methods Mol Biol* 2013, **939:**97-113.

7. Pahikkala T, Airola A, Pietila S, Shakyawar S, Szwajda A, Tang J, Aittokallio T: **Toward more realistic drug-target interaction predictions.** *Brief Bioinform* 2015, **16:**325-337.

8. Laub AJ: *Matrix Analysis For Scientists And Engineers.* Society for Industrial and Applied Mathematics; 2004.

9. Nen M, Alpayd, Ethem N: **Multiple Kernel Learning Algorithms.** *Journal of Machine Learning Research* 2011, **12:**2211-2268.

10. Hue M, Riffle M, Vert JP, Noble WS: **Large-scale prediction of protein-protein interactions from structures.** *BMC Bioinformatics* 2010, **11:**144.

11. Kloft M, Brefeld U, Laskov P: **Non-sparse Multiple Kernel Learning.** *Nips Workshop on Kernel Learning Automatic Selection of Optimal Kernels* 2008, **7:**775--782.
